# Altered expression of complement regulatory proteins CD35 and CD59 in acute lymphoblastic and acute myeloblastic leukemia suggests a context-based pattern

**DOI:** 10.1007/s12672-026-04818-3

**Published:** 2026-04-02

**Authors:** Reem Hossam EL-Din Ahmed, Menatalla Mostafa Mahmoud, Mohamed Ahmed El-Azizi, Noha Samir Farag

**Affiliations:** Microbiology and Immunology Department, Faculty of Pharmacy and Biotechnology, German University in Cairo, Cairo, Egypt

**Keywords:** Complement, mCRP, CD35, CD59, Acute leukemia, ALL, AML

## Abstract

The complement system is a vital part of the innate immune defense, playing a key role in clearing immune complexes; however, its activity must be carefully regulated to avoid damage to healthy cells. Complement plays a dual regulatory role in carcinogenesis, affecting the immune response outcomes. Membrane-bound complement regulators (mCRPs) include CD59 and CD35; where CD59 inhibits MAC formation and CD35 binds C3b/C4b for immune clearance. The current study aims to assess post-transcriptional expression levels of complement regulatory proteins CD35 and CD59 in acute leukemia patients. CD35 expression was significantly downregulated in acute leukemia in comparison to their normal counterparts at both transcriptional and protein levels (5.3-and 4-folds respectively), conversely CD59 expression was significantly increased (3 and 1.3 folds respectively). An HSB-2 acute leukemia cell model was established, and post-transcriptional silencing of CD35 and CD59 using shRNA was performed. MTT viability assays showed increased viability following CD35 silencing; conversely CD59 silencing caused a significant decrease in cell viability. The results suggest an altered pattern of mCRP expression on leukemic cells; where CD35 was downregulated, possibly to benefit from the complement system activation products in promoting carcinogenesis. Alternatively, leukemic cells might overexpress CD59 to enhance cell survival and escape MAC induced lysis. Our results suggest that abnormally altered expression of complement regulatory proteins (CRPs) could be a key mechanism employed by leukemic cells to evade destruction by the host's complement system to promote tumor progression, and indicate their potential implication as differentiation markers in acute leukemias.

## Introduction

The complement system is a crucial component of the innate immunity [1]. Complement proteins, which originate from both tumor cells and leaky blood vessels in the tumor microenvironment, contribute to inflammation and cell damage [2, 3]. These proteins circulate in an inactive form in the bloodstream [4] and become active to help the immune system respond to infections, injuries, and malignant cells [1, 5]. They are essential in removing immune complexes and apoptotic bodies as well [2]. The complement system can be activated via three pathways: the classical pathway (triggered by antigen–antibody complexes), the alternative pathway (activated by various surfaces), and the lectin pathway (initiated by mannose-binding lectins recognizing carbohydrate ligands on pathogens) [5]. However, uncontrolled activation of the complement system can lead to damage to the body's own cells. This process is carefully regulated by a range of regulatory proteins in plasma and on cell surfaces [6–8].

Complement regulatory proteins (CRPS) can be categorized into three main groups: (I) Fluid-phase proteins; factor H, C4BP, and factor I; (II) Cell-surface proteins; and (III) Membrane-integral proteins. These are embedded in the cell membrane and include CD35 (complement receptor 1 (CR1)), CD46 (membrane cofactor protein (MCP), CD55 (decay-accelerating factor (DAF)), and CD59 (protectin) [8,9].

CD35 is a transmembrane glycoprotein expressed on various cell types, including B cells, follicular dendritic cells [10,11], and others [12, 13]. It controls the complement system by assisting in the inactivation of C3b and C4b into iC3b and iC4b, respectively, with the help of factor I [14]. Additionally, CD35 inhibits the activation of the terminal complement complex by cleaving C3b [15] and accelerates the decay of C3 and C5 convertases, acting as both a co-factor and a decay-accelerating factor [16].

CD59 (Protectin) inhibits the formation of the membrane attack complex (MAC) by binding to C8α, thereby preventing C9 insertion [17]. Additionally, it modulates T-cell activity and is involved in various other cellular processes, including cell signaling, lipopolysaccharide (LPS) signaling, and apoptosis [3, 14, 18, 19].

Recent reports highlight the dual role of the complement system in modulating tumor microenvironment. Complement activation products, such as the anaphylatoxin C5a, were found to create an inflammatory microenvironment, which is associated with tumor formation, progression and transformation. Accordingly, recent studies suggest that complement activation can promote tumor growth instead of hindering it. As limited research has explored the impact of complement and its membrane-bound complement regulatory proteins (mCRPs) in shaping tumor microenvironment, this study aims to investigate the altered expression of CD35 and CD59 in Egyptian patients with acute leukemia and its impact on its pathogenesis.

## Methods

### Clinical specimen

Sixty-five peripheral blood samples were collected from Egyptian patients at the National Cancer Institute, Cairo (NCI). However, 39 samples (13 acute lymphoblastic leukemia samples and 26 acute myelogenous leukemia samples) were included in the study, while the rest were excluded as they did not meet the study's criteria. The age range was between 20 and 70 years. All cases were newly diagnosed and had not received chemotherapy or radiotherapy. Sixteen blood samples were also collected from healthy matching controls. The clinical characteristics of the enrolled patients, including age, sex, and leukemia subtype, are detailed in Table 1. The study was approved by the Ethics Committee of the German University in Cairo (MIB-2020-07-MEA) and conducted in accordance with the Declaration of Helsinki and the regulations of the National Cancer Institute, Cairo. An informed consent to participate in the study was obtained from all participants.

**Table 1:** Characterization of Acute leukemia patients

Sample name	Gender	Diagnosis	Age
1	Male	T-ALL	20
2	Female	AML-M3	68
3	Female	AML-M3	30
4	Male	T-ALL	30
5	Female	T-ALL	43
6	Female	AML-M4	27
7	Female	AML-M4	52
8	Male	AML-M4	31
9	Female	Pre B-ALL	20
10	Male	AML-M2	43
11	Male	AML-M2	32
12	Male	C-ALL	40
13	Female	AML-M1	45
14	Male	ALL	62
15	Male	Pre-B-ALL	54
16	Male	AML	56
17	Male	ALL	43
18	Male	AML-M0	60
19	Male	AML-5	60
20	Male	AML-M3	25
21	Female	AML-2	56
22	Male	AML-M2	35
23	Male	AML-M3	60
24	Male	AML-M1	50
25	Male	AML-M3	55
26	Male	AML-M2	42
27	Male	ALL	25
28	Male	ALL	29
29	Male	AML-M3	30
30	Male	AML-M6	44
31	Male	ALL	22
32	Female	T-ALL	30
33	Female	AML-M3	40
34	Female	AML-M2	30
35	Female	AML-M1	40
36	Female	AML-M2	50
37	Female	AML-M1	34
38	Female	AML	23
39	Female	ALL	26

### Total genomic RNA extraction and quantitative real-time PCR

Total genomic RNA was isolated following the Genomic Medicine Biorepository (GMB) standard protocol. In brief, red blood cells (RBCs) were lysed using ACK lysis buffer (Thermo Fisher Scientific, USA). After cell separation, TRIzol reagent was added, and RNA extraction was performed through phase separation with chloroform. The RNA was then precipitated using isopropanol, washed with ethanol, and dissolved in RNase-free water before being stored at − 80 °C. RNA quantity and purity were determined using a NanoDrop spectrophotometer, while integrity was verified by 1% agarose gel electrophoresis.

The extracted RNA was reverse-transcribed into complementary DNA (cDNA) using the High-Capacity cDNA Reverse Transcription Kit (Thermo Fisher Scientific, USA) in a Biometra T-Personal Thermocycler. Gene expression analysis was carried out by quantitative reverse transcription polymerase chain reaction (qRT-PCR) using the StepOnePlus™ Real-Time PCR System (Applied Biosystems, USA) and TaqMan® gene expression assays specific for CD35 and CD59 (ThermoFisher scientific, USA), with the reference gene β-actin used as an internal control. Relative expression levels were calculated using the 2^ − ΔΔCt^ method.

### Fluorescence-*a*ctivated cell sorting (FACS)

Peripheral blood samples were collected in heparinized tubes and stored at room temperature. All samples were processed within 6 h of collection. A 200 μL aliquot of whole blood was transferred into a flow cytometry tube, followed by the addition of 3 mL of ACK lysis buffer to remove red blood cells. The mixture was incubated for 5 min at room temperature. Lysis was terminated by adding 10 mL of cell staining buffer, after which the samples were centrifuged at 300×*g* for 5 min at room temperature. The supernatant was discarded, and the washing step was repeated.

The resulting cell pellet was resuspended in cell staining buffer, and the cell concentration was adjusted to 1 × 10⁷ cells/mL. For immunostaining, cells were incubated with PE-conjugated anti-CD35 and FITC-conjugated anti-CD59 monoclonal antibodies (Thermo Fisher Scientific, USA) according to the manufacturer’s instructions. Expression levels of CD35 and CD59 were analyzed by flow cytometry (FACS).

### Cell culture

Acute lymphoblastic leukemia HSB-2 cell line (kindly provided by Prof. Hassan Adwan, Ruprecht Karl University of Heidelberg, Germany) suspensions were maintained in RPMI -1640 medium supplemented with l-glutamine, 10% FBS (Biowest, USA), 1% non-essential amino acids, 1 mM sodium pyruvate, and 50 µg/mL penicillin–streptomycin (Lonza, Belgium). Cells were cultured in T75 cm^2^ flasks and incubated in 5% CO_2_ at 37 °C.

### Post transcriptional silencing of CD35 and CD59 using ShRNA

HSB-2 cells were cultured in T75 cm^2^ flasks until 70–80% confluency. A 1:2 mixture of cell suspension and Trypan Blue (Fluka Sigma, Germany) was prepared, and 10 µL was loaded into a hemocytometer for cell counting. For experiments, 5 × 10^5^ cells/well in 500 µL serum-free medium were seeded and incubated at 37 °C in 5% CO₂ for 24 h.

Cells were transfected with shRNA plasmids targeting **CD35** or **CD59** (Santa Cruz Biotechnology, USA) using polyethylenimine (PEI) were used to achieve stable silencing. A mock shRNA plasmid was used as control. Plasmid–PEI complexes were incubated for 30 min in RPMI medium before addition to cells. After 24 h, transfected cells were selected with puromycin.

Transfection efficiency and gene silencing were confirmed 24 h later by flow cytometry (Coulter® Epics XL-MCL™). Expression of CD35 and CD59 was evaluated using PE-conjugated anti-CD35 and FITC-conjugated anti-CD59 monoclonal antibodies.

### MTT cell viability assay

Cell viability after CD35 and CD59 gene silencing was assessed using the MTT assay. Transfected cells were incubated overnight at 37 °C with 5% CO₂, after which the medium was replaced with 50 μL fresh medium and 50 μL MTT solution (5 mg/mL in PBS). Following 6 h of incubation, formazan crystals were dissolved in 1 mL DMSO, and absorbance was measured at 595 nm using a Victor 1420 microplate reader.

### Statistical analysis

Statistical analyses were conducted using GraphPad Prism software (Version 5, GraphPad Software, USA). Data are expressed as mean ± standard deviation (SD). Differences between groups were analyzed using one-way ANOVA and unpaired *t*-tests. A *p*-value < 0.05 was considered statistically significant.

## Results and discussion

### Assessment of CD35 and CD59 expression level in ALL and AML patients by q RT-PCR

**Fig. 1:**
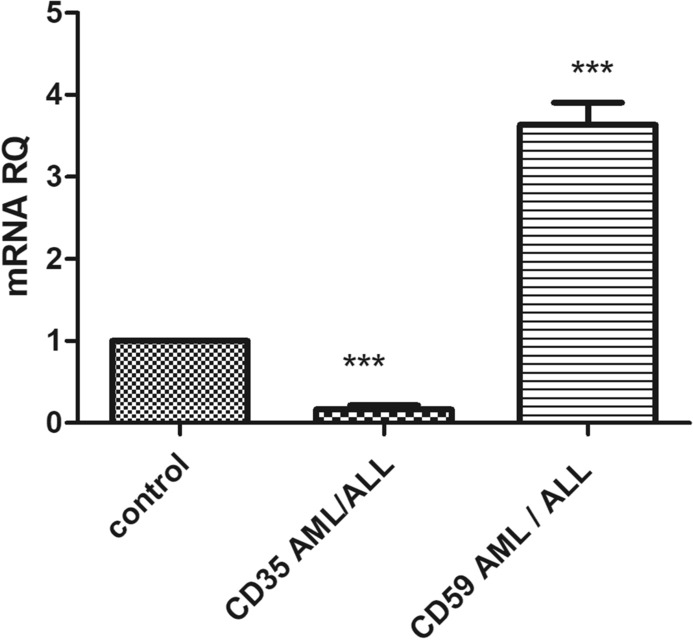
Expression of CD35 and CD59 in acute leukemia patients (both ALL and AML) compared to healthy controls. CD35 and CD59 mRNA expression levels were measured using qRT-PCR in acute leukemia patients, normalized to the housekeeping β-actin genes, and results were compared to CD35 and CD59 mRNA expression level in healthy controls

**Fig. 2:**
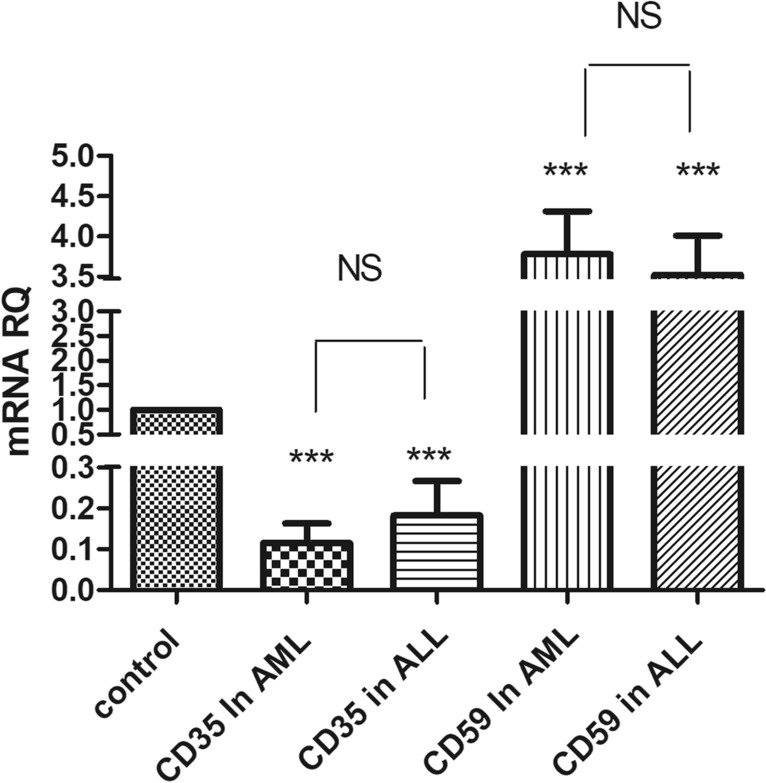
Expression of CD35 and CD59 in ALL and AML compared to healthy controls. CD35 and CD59 mRNA expression levels were measured using qRT-PCR in acute leukemia patients, normalized to the housekeeping β-actin genes, and results were compared to both CD35 and CD59 mRNA expression level in healthy controls

Quantitative real time qRT-PCR was carried out to determine the steady state levels of mRNA transcripts of both mCRPs in 39 acute leukemia patients from both genders, in comparison with healthy subjects**.** Figure 1 showed that CD35 expression was significantly down regulated in acute leukemia patients compared to healthy individuals by 5.3 folds (p < 0.0001). As shown in Fig. 2, AML patients showed an 8.6-fold decrease (p < 0.0001), while ALL patients showed a 4.2-fold decrease (p < 0.0001) in CD35 expression levels compared to healthy controls.

On the other hand, CD59 expression was significantly elevated in acute leukemia by threefold (p < 0.0001), as depicted in Fig. 1. Moreover, AML patients showed a 3.78‑fold increase (p < 0.0001), while ALL patients showed a 3.52‑fold increase (p < 0.0001) in CD59 expression levels compared to healthy controls, as shown in Fig. 2.

**Fig. 3:**
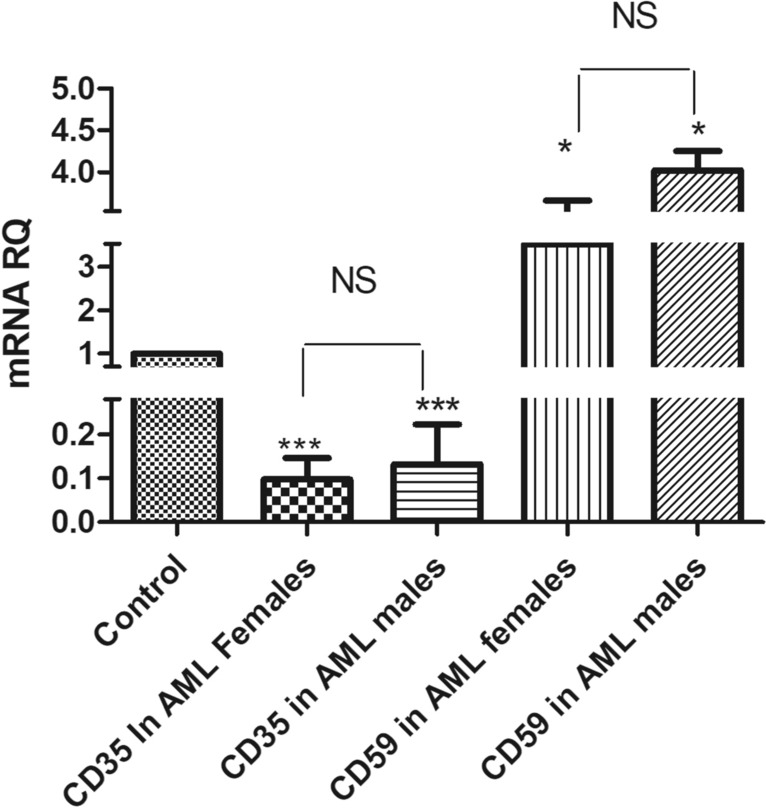
CD35 and CD59 mRNA expression levels based on gender differences in AML patients. CD35 and CD59 mRNA expression levels were measured using qRT-PCR in female and male AML patients, normalized to the housekeeping β-actin genes, and results were compared to both of CD35 and CD59 mRNA expression level in healthy controls

**Fig. 4:**
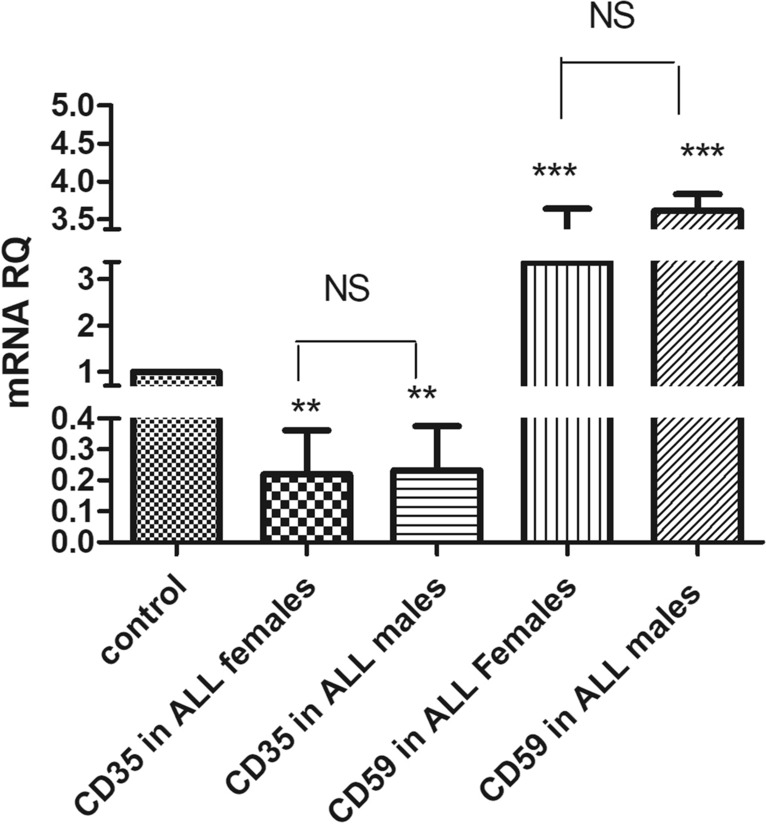
CD35 and CD59 mRNA expression levels based on gender differences in ALL patients. CD35 and CD59 mRNA expression levels were measured using qRT-PCR in ALL males and females, normalized to the housekeeping β-actin genes, and results were compared to both of CD35 and CD59 mRNA expression levels in healthy controls

The expression levels of CD35 and CD59 in males compared to female patients of AML and ALL were assessed as well to determine if gender discrepancy influenced these biomarkers expression. Both male and female patients exhibited a reduced CD35 expression compared to healthy controls. Specifically, males with AML showed a 7.5-fold reduction (p < 0.0001), while females with AML showed a tenfold reduction (p < 0.0001). Similarly, male ALL patients showed a fourfold decrease (p = 0.0049), and females ALL patients showed a 4.4-fold decrease (p = 0.049) as represented in Figs. 3 and 4. No significant difference in CD35 expression was observed between male and female patients in either AML or ALL.

On the other hand, results showed a marked upregulation of CD59 expression in both male and female patients with AML and ALL compared to healthy controls. In AML, CD59 expression increased approximately fourfold in male patients and 3.53-fold in female patients relative to controls. Similarly, both male and female ALL patients exhibited significant CD59 overexpression compared to healthy subjects, with males showing a 3.61-fold increase (p < 0.0001) and females a 3.37-fold increase (p < 0.0001). Overall, CD59 expression was slightly higher in male patients than in female patients in both leukemia types, as shown in Figs. 3 and 4.

The downregulation of CD35 in acute leukemia patients could suggest that the cancerous cells manipulate the complement expression and activation to its benefit. This can be further explained by many findings concerning the tumor promoting role of complement activation in the TME, role of complement components and anaphylatoxins (C3a and C5a) in the process of carcinogenesis and the role of complement component C1q. The complement component C1q, which is a ligand for CD35, is expressed in the stroma and vascular endothelium of various human malignancies [20]. The binding of C3a and C5a anaphylatoxins to their receptors boosts angiogenesis via the upregulated expression of growth factors such as basic fibroblast growth factor (bFGF) and vascular epithelial growth factor (VEGF) and the proliferation of endothelial cells is enhanced [21].

On the other hand, our results show that CD59 is upregulated in acute leukemia patients compared to healthy controls. One possible theory for the elevated expression of CD59 on surface of cancer cells is the inhibition of MAC formation [22, 23]. Elevated expression of CD59 was highly effective in protecting tumour cells from complement attack and it works synergistically with CD46 and CD55 [24]. CD59 also influences the activity of CD4 + T-cells by downregulating them to limit T-cell mediated immune attack. An earlier study showed that CD4 + T-cells freshly isolated from colorectal cancer (CRC) patients, which expressed CD25 and Fox p3, highly expressed CD59 more than CD4 + T-cells negative for these markers [25].

### Assessment of surface expression of CD35 and CD59 in peripheral blood by FACS

**Fig. 5:**
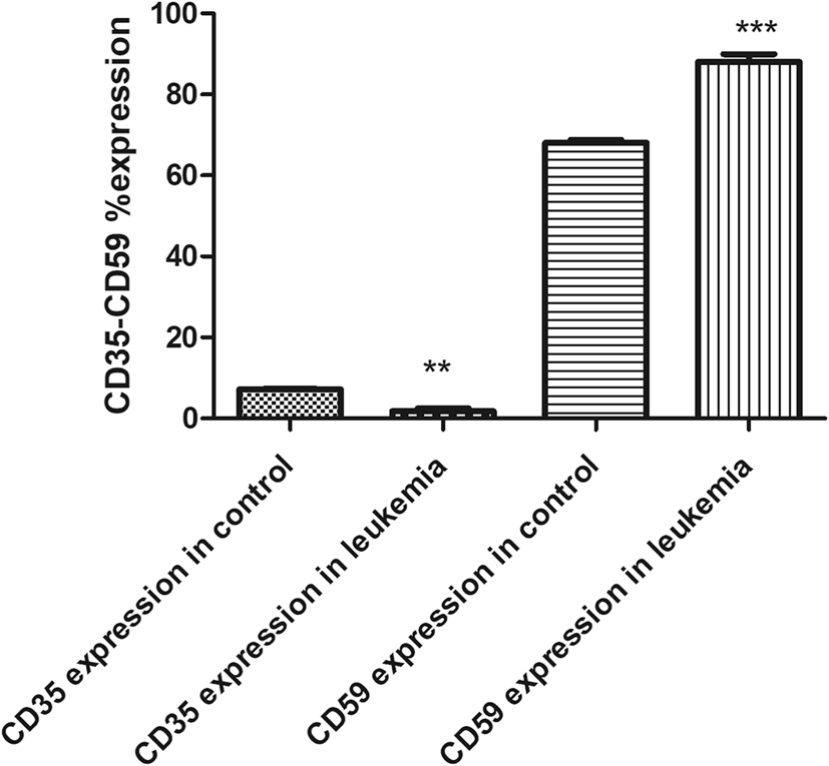
Percentage of CD35 and CD59 expression in acute leukaemia patients. FACS charts showed that expression of CD35 in leukemic patients was reduced by 76.1% when compared to healthy samples, however CD59 expression increased by 20%. *p < 0.05; **p < 0.01, ***p < 0.001

**Fig. 6:**
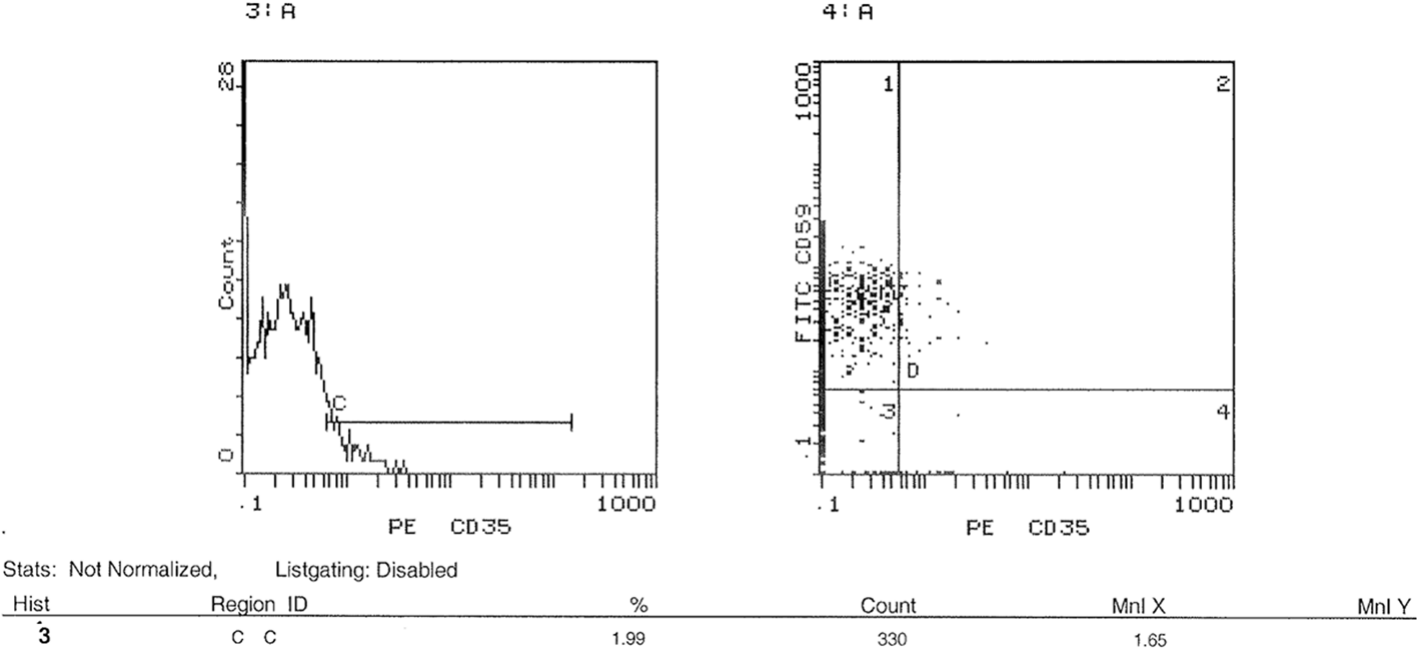
CD35 expression levels on cell surfaces using FACS. Cells from blood samples of acute leukemia patients and healthy controls were isolated and analyzed for CD35 expression using flow cytometry (FACS). **A** and **B** reveal that 1.99% of cells from acute leukemia patients expressed CD35, as indicated by their presence in the D4 quadrant

**Fig. 7:**
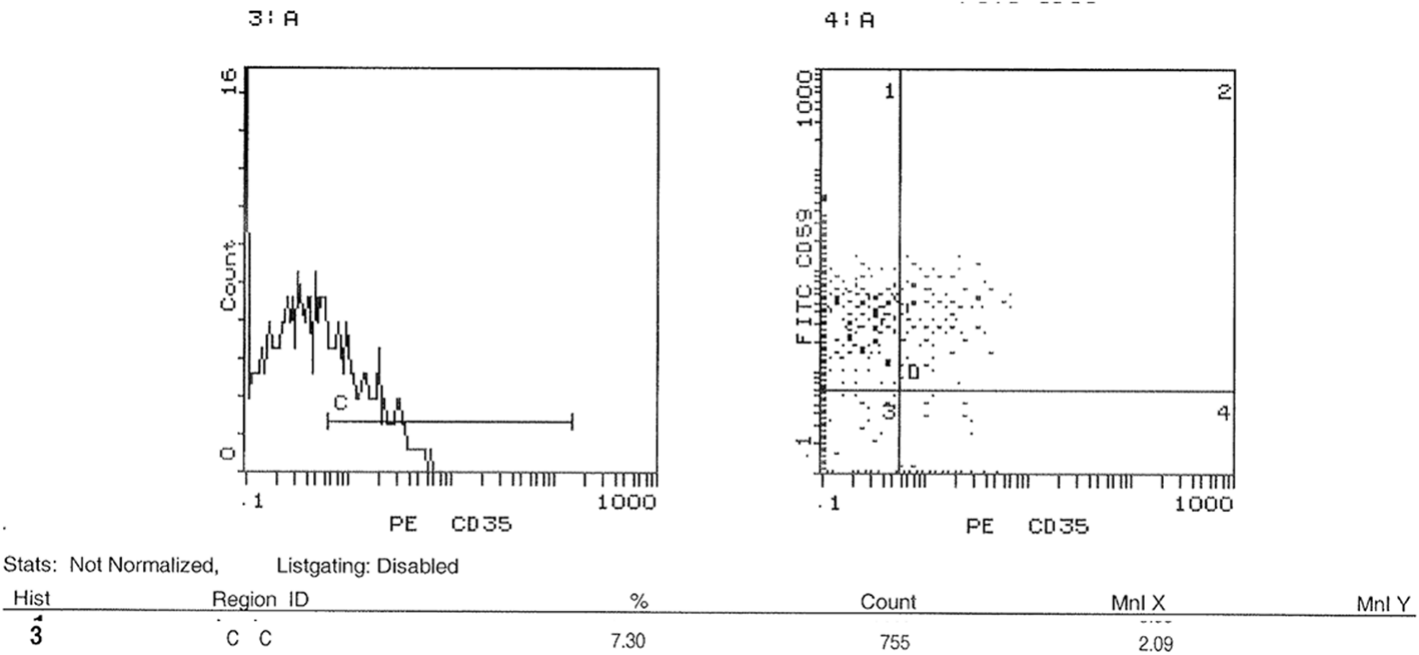
CD35 expression levels on cell surfaces using FACS. Cells from blood samples of acute leukemia patients and healthy controls were isolated and analyzed for CD35 expression using flow cytometry (FACS). **A** and **B** show that a higher percentage (7.3%) of cells from healthy controls expressed CD35, also localized to the D4 quadrant

**Fig. 8:**
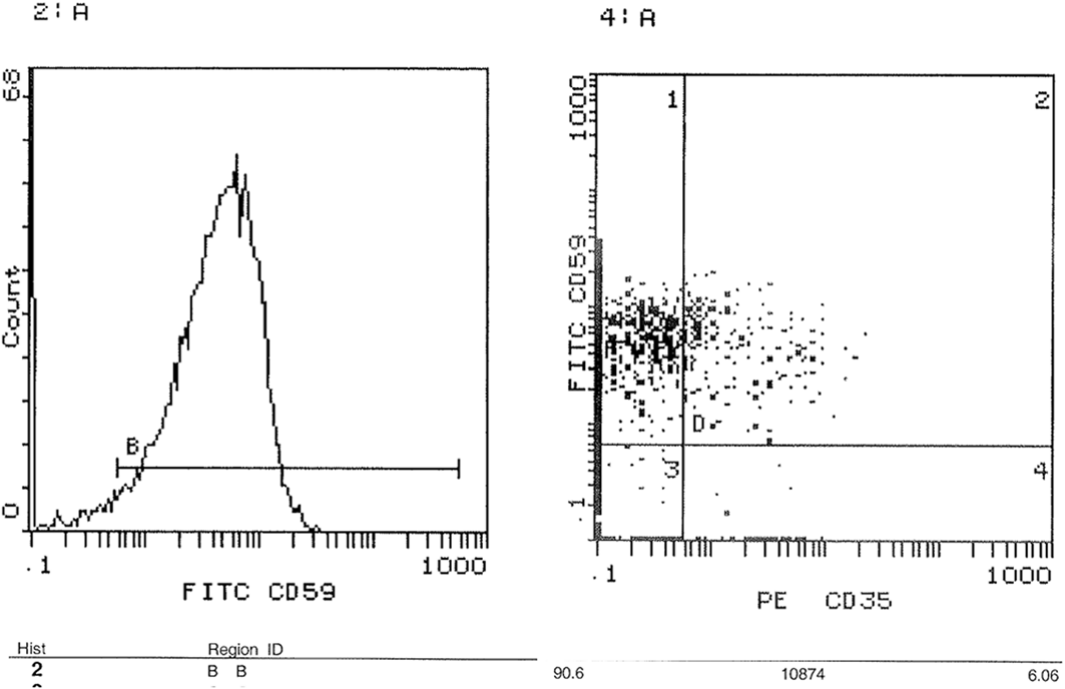
CD59 expression levels on cell surfaces using FACS. Cells from blood samples of acute leukemia patients and healthy controls were isolated and analyzed for CD59 expression using flow cytometry (FACS). **A** and **B** show that 90.6% of cells from acute leukemia patients expressed CD59, as indicated by their position in the D4 quadrant

**Fig. 9:**
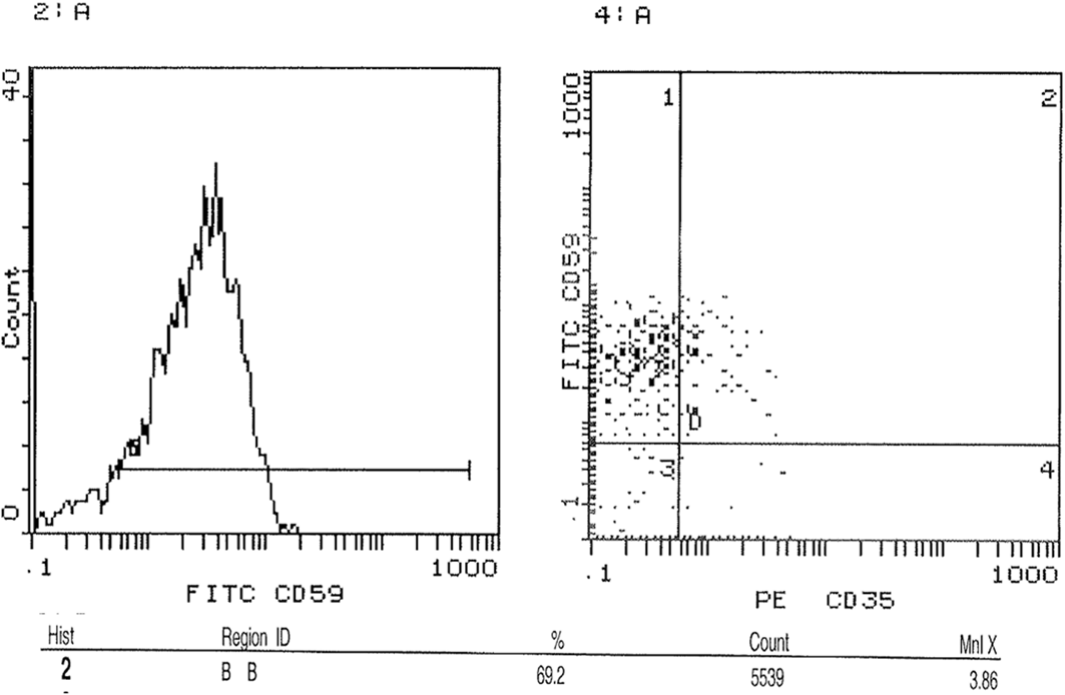
CD59 expression levels on cell surfaces using FACS. Cells from blood samples of acute leukemia patients and healthy controls were isolated and analyzed for CD59 expression using flow cytometry (FACS). **A** and **B** show that 69.2% of cells from healthy controls expressed CD59, as indicated by their position in the D4 quadrant

FACS was performed to investigate the expression level of CD 35 and CD59 proteins on cell surfaces. Results showed a significant decrease in the expression of CD35 in acute leukemia patients compared to healthy controls. The average CD35 expression in leukemia patients was 1.7%, compared to 7.1% in healthy controls. This represents a 76.1% decrease in CD35 expression in leukemia patients (p < 0.001) as shown in Fig. 5. The expression profile of representative samples, in which CD35 expression was 1.99% in the acute leukemia case and 7.3% in the healthy control (Figs. 6 and 7), was consistent with these findings. FACS analysis confirmed these findings, visualizing the reduced CD35 expression in leukemia patient samples. On the other hand**,** FACS analysis also confirmed significantly higher CD59 expression in leukemic patients (88%) compared to healthy controls (68%) (p < 0.0006), Fig. 5. This is further illustrated by the results showing that the average CD59 expression in leukaemia patients was 88%, compared to 66% in healthy controls. The expression profile of representative samples showed that CD59 expression was 90.6% in the acute leukaemia case and 69.2% in the healthy control (Figs. 8 and 9).

The findings of our study align with previous research on both haematological malignancies and solid tumors that correlate the significant down regulation of CD35 on erythrocytes in patients with various malignancies, to the vulnerability of red blood cells to proteinases, which is often associated with cancers and inflammatory conditions [26, 27]. The damage and breakdown of neutrophils in cancerous conditions release and activate numerous protein-degrading enzymes. This can lead to local inactivation of proteinase inhibitors and the cleavage of CD35 by granule enzymes.

On the other hand, CD59 plays a crucial role in protecting tumor cells from complement-mediated killing by inhibiting the formation of the membrane attack complex (MAC). Elevated expression of CD59 on leukemic cells has been associated with poor prognosis, chemotherapy resistance, and enhanced survival of leukemia blasts. Studies have confirmed that CD59 overexpression contributes to leukemic cell survival by preventing complement activation, as well as by reducing apoptosis and enhancing cell proliferation. This protective effect extends to tumor cells in other malignancies, such as non-small cell lung cancer (NSCLC), where CD59 overexpression inhibits complement-mediated lysis [22, 28].

### Flow cytometric analysis of transfection success post-knockdown of CD35 and CD59 in HSB-2 cell line

**Fig. 10:**
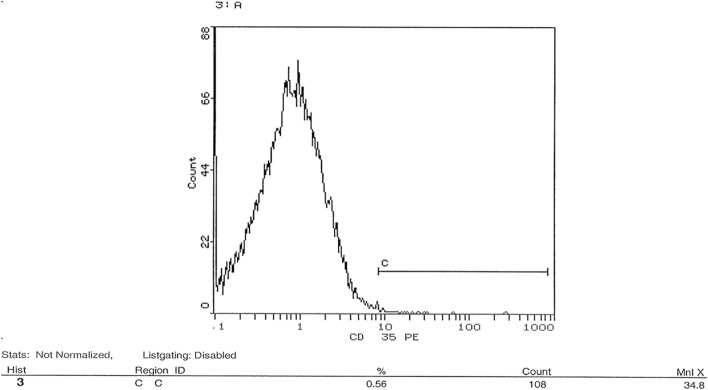
CD35 expression on CD35 shRNA untransfected cells. Cells were analyzed for CD35 expression using flow cytometry. Data from representative experiment samples of a CD35 untransfected cells were analyzed. Flow cytometry charts indicate an average CD35 expression level of 0.56% on untransfected cells

**Fig. 11:**
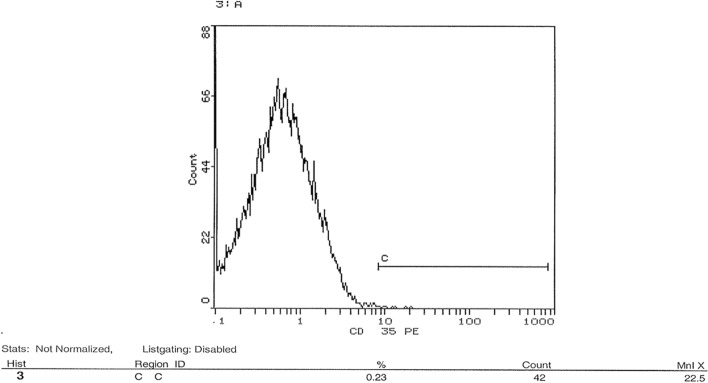
CD35 expression on CD35 shRNA transfected cells. Cells were analyzed for CD35 expression using flow cytometry. Data from representative experiment samples of a CD35 transfected cells were analyzed. Flow cytometry charts indicate an average CD35 expression level of 0.23% on transfected cells

**Fig. 12:**
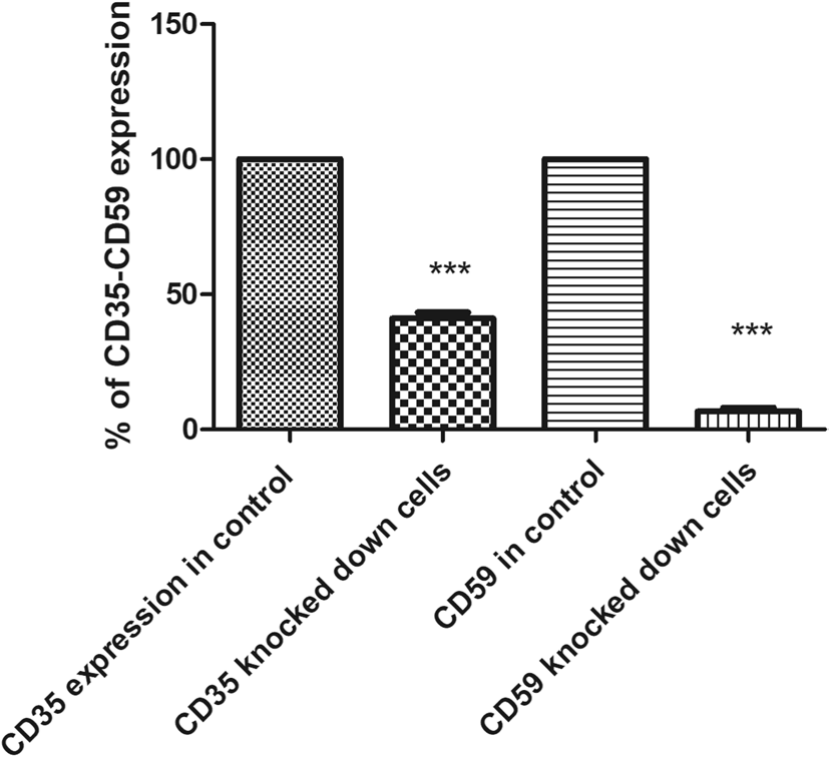
Determination of shRNA-mediated knockdown of CD35 and CD59 mCRP expression on HSB-2 cells compared to untransfected controls. Reducing CD35 gene expression in transfected cells decreased CD35 expression by 59% (p < 0.0001). Similarly, reducing CD59 gene expression led to a 98.95% decrease in CD59 expression levels (p < 0.001)

**Fig. 13:**
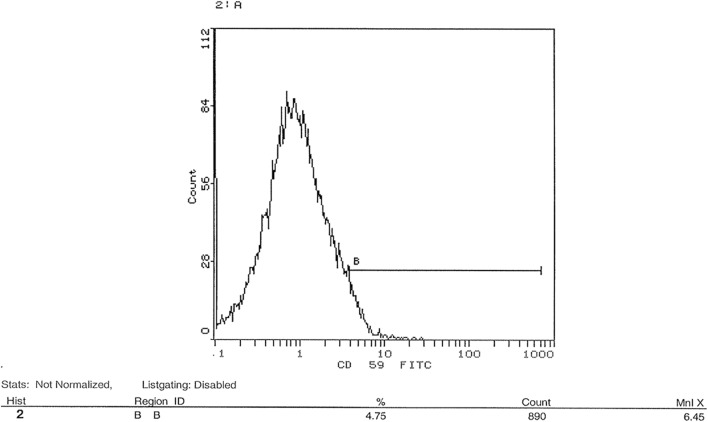
CD59 expression on CD59 shRNA untransfected cells. Cells were analyzed for CD59 expression by flow cytometry. Data were obtained from representative experiment samples of a CD59 un-transfected cells. Flow cytometry charts showed an average of CD59 expression level of 4.75% on untransfected cells

**Fig. 14:**
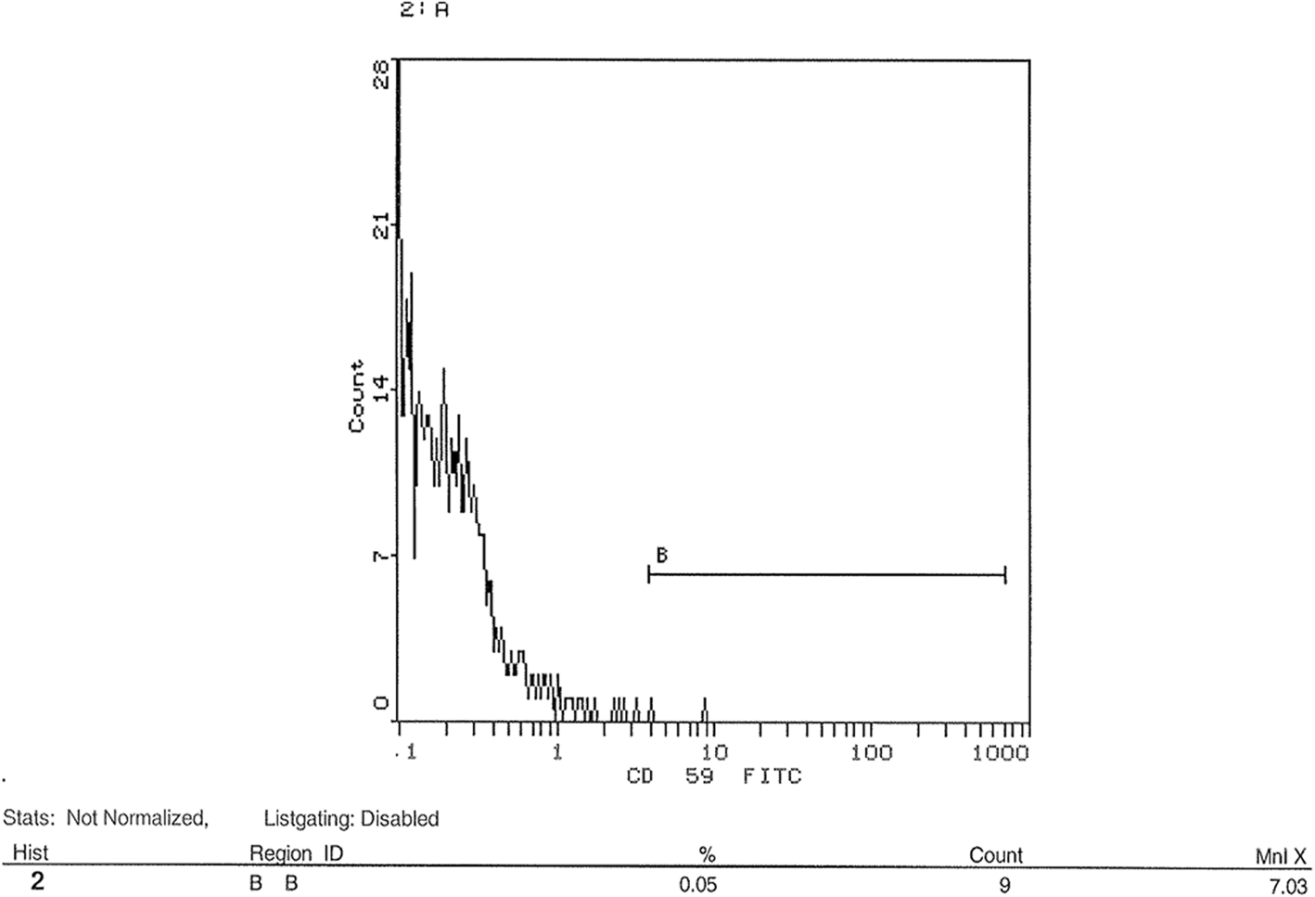
CD59 expression on CD59 shRNA transfected cells. Cells were analyzed for CD59 expression by flow cytometry. Data were obtained from representative experiment of CD59 transfected cells. Flow cytometry charts showed an average of CD59 expression level of 0.05% on cells transfected

shRNA was used in this study to knockdown gene expression of CD35 and CD59 in HSB-2, an ALL-cell line. Expression level of CD35 and CD59 was measured by flow cytometry using CD35 and CD59 monoclonal antibodies. Figure 10 showed that un-transfected cells expressed CD35 at a level of 0.56%. Figure 11 indicated that transfected cells expressed CD35 at a level of 0.23%. Silencing CD35 in transfected cells using a CD35 shRNA silencing plasmid resulted in a significant 59% reduction in CD35 expression compared to mock cells, as depicted in Fig. 12 (p < 0.0001). On the other hand, Fig. 13 showed un-transfected cells expressed CD59 at a level of 4.75%. Figure 14 indicated that transfected cells expressed CD59 at a level of 0.05%.

CD59 expression was significantly reduced by 98.95% in cells transfected with CD59 shRNA silencing plasmid compared to control cells (p < 0.001), as illustrated in Fig. 12.

### Cellular viability assays of HSB-2 post CD35 and CD59 knockdown

**Fig. 15:**
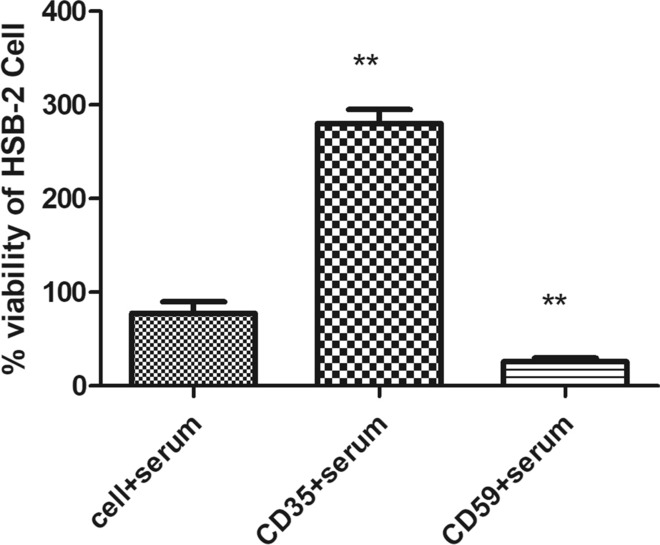
Acute Lymphoblastic Leukaemia cells viability after silencing of CD35 and CD59 mCRPs. The viability of cells transfected with plasmids targeting CD35 and CD59 was compared to untreated cells. Cell viability was measured using the MTT assay in the presence of normal human serum as a complement source. Statistic'ally significant differences were observed between the groups (*p < 0.05; **p < 0.01, ***p < 0.0011)

Cell viability was assessed using an MTT assay in the presence of normal human serum as a source of complement to evaluate response of cancer cells to complement components after successfully silencing CD35 and CD59. Following silencing of CD35 expression using shRNA in HSB-2 cells, cellular viability increased significantly by 4 folds (p = 0.0048) when cultured in presence of complement proteins, as shown in Fig. 15. This suggests that CD35 silencing can protect cells from complement-mediated lysis and promotes leukemic blast survival and proliferation. Furthermore, it was observed that cell viability was significantly decreased by 71.8% upon silencing CD59 (p < 0.0018), suggesting that CD59 is a necessary membrane-bound protein for leukemic cells to survive. Its expression could contribute to invasiveness and metastases of those cells.

The interplay between CD35 and CD59 in leukemia is likely linked to their roles in modulating the complement system. Downregulation of CD35 results in increased levels of complement anaphylatoxins (C3a and C5a), which promote inflammation and carcinogenesis. Moreover, CD59 upregulation allows leukemia cells to evade immune surveillance by preventing complement-mediated cell destruction, potentially through interactions with immune cells like Tregs and myeloid-derived suppressor cells (MDSCs) in the tumor microenvironment (TME) [29].

Together, these findings suggest that both CD35 and CD59 play pivotal roles in leukemia progression by manipulating the complement system. The downregulation of CD35 allows leukemia cells to benefit from enhanced complement activation, while the upregulation of CD59 protects these cells from complement-mediated destruction, promoting survival and contributing to chemotherapy resistance. This dual role of complement regulators highlights the complexity of the complement system in cancer, where it can either suppress tumor growth or promote it depending on the context, and underscores the potential for targeting these proteins as therapeutic strategies in leukemia and other malignancies.

## Conclusion and future perspectives

The results suggested that CD35 downregulation increased cellular viability in the presence of complement proteins, supporting the hypothesis that complement cascade activation could be important for leukemic blasts proliferation. However, CD59 expression was necessary for tumours to escape complement surveillance by inhibiting MAC formation, decreasing T-cell activity and decreasing apoptosis, thus, aiding tumour growth to thrive. It was observed that there is a general trend of upregulation of CD59 expression in AML and ALL patients and decreased cell viability after CD59 gene silencing by shRNA plasmids suggesting that the complement system may have a function in creating an amiable environment for cancer to grow and progress.

The current study is one of very few studies that examined complement activity in haematological malignancies and the first one to study its role in Egyptian patients.

Due to the numerous roles that complement plays in several diseases, further investigation is needed regarding its direct implication in regulating tumour specific setting. Additionally, other studies on other types of cancer should be carried out, such as chronic myeloid leukemia, chronic lymphocytic leukemia, lymphomas, and other forms of cancer as well. Further investigation can be performed by fellow colleagues to assess the effect of combining CD35 and CD59 with other mCRPs on carcinogenesis and the various genetic polymorphisms in the CD35 gene.

## Data Availability

Data are available upon request addressed to [ronaawad3@gmail.com](mailto:ronaawad3@gmail.com) . The datasets analysed during the current study are available in the following repositories: - CD59 gene and protein - NCBI Gene ID: 966 - OMIM (MIM): 107271 - Ensembl: ENSG00000085063 - CR1 (CD35) gene and protein - NCBI Gene ID: 1378 - OMIM (MIM): 120620 - Ensembl: ENSG00000203710 Gene expression analyses were performed using commercially available TaqMan Gene Expression Assays: - CD59 TaqMan Assay (Hs00174141_m1) - Forward primer: 5′-TGCAATTTCAACGACGTCACA-3′ - Reverse primer: 5′-GAAATGGAGTCACCAGCAGAAGA-3′ - CD35 (CR1) TaqMan Assay (Hs00559348_m1) - Forward primer: 5′-TAGGTGTCAGCCTGGCTTTGTC-3′ - Reverse primer: 5′-GACATCTGGAGGTGGCTGACAT-3′ Gene silencing experiments were conducted using commercially available short hairpin interfering RNA constructs: - CD59 shRNA (sc-37249-SH) - Target sequence: 5′-TGAGCTAACGTACTACTACTGC-3′ - CD35 (CR1) shRNA (sc-29994-SH) - Target sequence: 5′-GGACATCAAGTGGCTAAAT-3′ All reagents are commercially available from Thermo Fisher Scientific and Santa Cruz Biotechnology. No novel primary sequence datasets were generated in this study requiring deposition in sequence repositories..
